# New Paradigms in Pharmaceutical Development

**DOI:** 10.3390/life12091433

**Published:** 2022-09-15

**Authors:** Ramón Cacabelos

**Affiliations:** International Center of Neuroscience and Genomic Medicine, EuroEspes Biomedical Research Center, 15165 Corunna, Spain; rcacabelos@euroespes.com

The main health problems in developed countries are cardiovascular diseases (25–30%), cancer (20–25%) and nervous system disorders (10–15%), which globally account for more than 80% of the morbidity and mortality in the general population. Infectious diseases, which were prevalent in developing countries in the immediate past, have gained unprecedented importance in our lives during the last three years with the emergence of COVID-19, as a result of pandemic infection with the SARS-CoV-2 virus. This pandemic, which paralyzed society and the world economy, also caused serious disruptions to pharmaceutical development programs focusing on other non-infectious pathologies. However, COVID-19 has reminded advanced societies that viruses also exist and can be lethal when allowed to circulate unchecked. As the industry transforms according to fashions or interests, it is not strange to observe that many pharmaceutical development programs in multinational arenas have turned towards antiviral treatments; however, this trend must change and, in fact, it is already changing. COVID-19 arrived loaded with enigmas and is leaving loaded with contradictions.

Despite the great advances that have taken place during the last decade in relation to the etiopathogenesis of heart disease and cancer or brain problems, there have been no great therapeutic discoveries that have dramatically revolutionized the ways of treating these prevalent pathologies. Although the prevalence of some of these diseases has decreased, this cannot be attributed to pharmacological improvements, but to the implementation of prophylactic programs. The situation is especially dramatic in the case of neurodegenerative diseases, which lack curative treatments and constitute a cause of great disability and societal cost.

In recent years, the scientific community has been able to influence the evidence suggesting that all chronic diseases have a course of pathogenic implantation across decades. By the time a disease of adulthood or old age manifests itself clinically, it has actually been destroying the body for many years and, when diagnosed, the consolidated damage is responsible for the symptoms. The symptomatic treatments on which 80% of current pharmacology is based are not negligible and have managed to mitigate pain, discomfort, fears, insecurity and physical and mental disability, but they lack etiopathogenic efficacy. Therefore, the future of pharmaceutical development lies in the need to discover anti-pathogenic treatments that can enable the implementation of preventive programs that are administered years before the disease manifests. To make this possible, it is also necessary to identify risk biomarkers that enable the prediction of the risk of being affected by a disease years before its clinical expression.

Another unfortunate finding of recent decades is the verification of the argument that the misuse and abuse of drugs are becoming the third key health problem in developed countries. Equally obvious is the finding that the same drug does not have the same effect on all people. In order to personalize treatments, it is necessary to apply the resources provided by genomic medicine. More than 80% of adult pathologies are polygenic and multifactorial, where the genome interacts with the environment to unbalance the individual’s health in favor of disease. In this genome–environment dialogue, epigenetics plays a fundamental role. A total of 100% of the pathologies present epigenetic aberrations that affect the global methylation of DNA, the disposition of nuclear chromatin and histones, and the dysregulation of the gene expression caused by micro-RNAs. The important fact to note about epigenetics, whose defects are transmitted from generation to generation, is that the lack of damage or alterations in the structure of DNA makes it a reversible process, the reversal of which can be achieved with drugs, nutritional strategies and modifications of the deleterious environment. Consequently, epigenetic drugs (EpiDrugs) open up a new prophylactic and therapeutic horizon that, potentially, can be valid for any human pathology. Unfortunately, these drugs are only just being approved for cancer treatment, and most anti-tumor EpiDrugs are quite toxic. However, new biotechnological products will make their way into the field of medical epigenetics for prophylactic and therapeutic purposes.

New research in pharmaceutical development has to import resources from medical genomics. Understanding predictive and diagnostic genomics would help to uncover new forms of effective therapeutic interventions, from gene therapy to epigenetic, transcriptomic, proteomic and metabolomic treatments.

The “one drug–one receptor” theory is a historical naivety. No drug, no matter how defined its molecular structure is, acts through a single mechanism of action. Most drugs fit better with the concept of a “dirty” mechanism, with various molecular anchor points and multi-purpose mechanisms of action. It is just as naïve to believe that a single drug will be enough to treat a complex disease.

Even most monogenic diseases require multifactorial treatments, when possible, to treat them at the molecular level. Moreover, in the elderly population, it is difficult to find patients with only one problem. Among patients aged from 50 to 65 years, 30% have more than one disease, and above the age of 70, more than 80% of the population has pluri-pathology treated with polypharmacy. Patients with a degenerative disease take 6 to 12 drugs or more and are victims of more than three concomitant diseases that require medication.

In this sense, pharmacogenetics comes to the aid of pharmaceutical science in order to develop new drugs of a high efficacy and low toxicity. The pharmacogenetic machinery includes pathogenic genes, associated with the pathology of a specific disease; mechanistic genes, related to the mechanism of action of a specific drug; metabolic genes, encoding the enzymes responsible for the metabolism of drugs in the liver and tissues and their elimination by the kidneys, bile, feces and waste fluids; transporter genes, which encode the transporter proteins responsible for whether or not a drug can access its therapeutic target; and pleiotropic genes, which participate in a multitude of metabolomic pathways that are altered by the disease. The consideration of these operational genetic clusters in the processing of any drug, when treating any disease, is of incalculable value for the acceleration of the process of development of new pharmaceutical specialties and new bioproducts.

Pharmacogenetics can help us, incontrovertibly, to develop more effective and less toxic drugs, to reduce development costs and, therefore, the final cost of the pharmaceutical product that has to be assumed by the patient or by state funds, and—in short—to optimize the therapeutic resources available today, as well as those for new future developments.

Carrying out the task of transforming the teleological approach to pharmaceutical science requires a change in the paradigm that dominates current pharmaceutical development strategies. Moreover, the paradigm shift demands a change in mentality among the scientific community, in the pharmaceutical industry, in the regulatory bodies of drug approval, in prescribing physicians and in users. The new pharmaceutical science needs to start thinking about developing drugs that can preserve health instead of investing all resources in replicating drugs for the repair or attenuation of the symptoms of the disease.

